# Cytogenetic profile of patients with Down syndrome in southern Brazil

**DOI:** 10.1590/1516-3180.2014.1324765

**Published:** 2014-05-28

**Authors:** Patrícia Trevisan, Felipe Nora de Moraes, Vinicius Freitas de Mattos, Carla Graziadio, Rafael Fabiano Machado Rosa, Giorgio Adriano Paskulin, Paulo Ricardo Gazzola Zen

**Affiliations:** I BPharm. Postgraduate Student, Postgraduate Program on Pathology, Universidade Federal de Ciências da Saúde de Porto Alegre (UFCSPA), Porto Alegre, Rio Grande do Sul, Brazil; II Medical Student, Universidade Federal de Ciências da Saúde de Porto Alegre (UFCSPA), Porto Alegre, Rio Grande do Sul, Brazil; III MD. Clinical Geneticist, Universidade Federal de Ciências da Saúde de Porto Alegre (UFCSPA), and Complexo Hospitalar Santa Casa de Porto Alegre (CHSCPA), Porto Alegre, Rio Grande do Sul, Brazil; IV MD. Assistant Professor of Clinical Genetics, Universidade Federal de Ciências da Saúde de Porto Alegre (UFCSPA), and Clinical Geneticist, Universidade Federal de Ciências da Saúde de Porto Alegre (UFCSPA) and Complexo Hospitalar Santa Casa de Porto Alegre (CHSCPA), Porto Alegre, Rio Grande do Sul, Brazil; V MD, PhD. Clinical Geneticist, Universidade Federal de Ciências da Saúde de Porto Alegre (UFCSPA) and Complexo Hospitalar Santa Casa de Porto Alegre (CHSCPA), Porto Alegre, Rio Grande do Sul, Brazil; VI PhD. Associate Professor of Clinical Genetics and Coordinator of the Postgraduate Program on Pathology, and Clinical Geneticist, Universidade Federal de Ciências da Saúde de Porto Alegre (UFCSPA) and Complexo Hospitalar Santa Casa de Porto Alegre (CHSCPA), Porto Alegre, Rio Grande do Sul, Brazil; VII PhD. Adjunct Professor of Clinical Genetics and Professor of the Postgraduate Program on Pathology, and Clinical Geneticist, Universidade Federal de Ciências da Saúde de Porto Alegre (UFCSPA) and Complexo Hospitalar Santa Casa de Porto Alegre (CHSCPA), Porto Alegre, Rio Grande do Sul, Brazil

Down syndrome is a common genetic disorder found in about one in every 800 live births. The diagnosis is normally confirmed through conventional karyotyping, through evidence of extra material from chromosome 21.^1^ The aim of our study was to investigate the cytogenetic findings of patients with Down syndrome who were diagnosed by a clinical genetics service in southern Brazil between 1975 and 2008. All karyotyping was performed in the same laboratory, using the G-bands by trypsin using Giemsa (GTG) technique. A mean of 22 cells per case were analyzed (range: 5 - 80).

Our sample comprised 644 patients (364 males and 280 females). Full trisomy of chromosome 21 was the predominant alteration observed in 598 of these patients (92.9%). Chromosomal abnormalities of structural type were observed in 26 patients (4%). These were composed of Robertsonian translocations involving chromosome 21 and chromosomes 14 (n = 12), 21 (n = 10), 15 (n = 1) and 22 (n = 1). In addition, two cases showed a reciprocal translocation, one of them involving chromosomes 21 and 5 and another with a complex translocation involving chromosomes 5, 21 and 22. Among the patients with structural chromosomal abnormalities, karyotyping analysis on the parents was performed in relation to 11 patients. Structurally balanced alterations were detected in five of them (45.5%). 

Mosaicism was identified in 20 patients (3.1%) and usually involved two cell lines: one normal and one with trisomy 21 (n = 15). However, two patients (10%) presented mosaicism involving three cell lines. Five cases (25%) showed a cell line with double aneuploidy, and four of them involved chromosomes 9, 14, 21 and X, respectively. The fifth patient presented an additional marker chromosome. Six patients (30%) among the 20 had mosaicism for trisomy 21 that was lower than 10%. The mean number of cells analyzed in these cases was 42 (the maximum was 80 cells; range: 15 - 80).

In our review of the literature, using the PubMed, SciELO and Lilacs databases, we did not identify any studies that evaluated the cytogenetic profile of patients with Down syndrome in Latin America. The frequencies of the different changes to chromosome 21 observed in our study were consistent with those described in the literature.^1^ ^-^ ^5^ Full trisomy of chromosome 21 was the main abnormality observed (84.8% to 97.7%). The structural abnormalities usually consist of Robertsonian translocations, involving chromosomes 14 and 21. Unusual structural abnormalities, as observed in two patients of our study, are considered quite rare.^2^ ^,^ ^5^ Detection of structural abnormalities is very important, since in these cases there is a need for parental karyotyping evaluation for assessment of future reproductive risk. The other uncommon chromosomal findings in our sample consisted of double aneuploidy and mosaicism presenting more than two cell lines.^5^

Thus, our study highlights the importance of performing karyotyping analysis on patients presenting a clinical diagnosis of Down syndrome, especially with a proper cell count, because of the low-grade mosaicism. This is important not only for confirming the diagnosis but also for determining the type of Down syndrome chromosomal alteration. This is essential for enabling appropriate genetic counseling for the family.

## References

[1] Murthy SK, Malhotra AK, Mani S (2007). Incidence of Down syndrome in Dubai, UAE. Med Princ Pract.

[2] Sheth F, Rao S, Desai M, Vin J, Sheth J (2007). Cytogenetic analysis of Down syndrome in Gujarat. Indian Pediatr.

[3] Mutton D, Alberman E, Hook EB (1996). Cytogenetic and epidemiological findings in Down syndrome, England and Wales 1989 to 1993. National Down Syndrome Cytogenetic Register and the Association of Clinical Cytogeneticists. J Med Genet.

[4] Jaouad IC, Cherkaoui Deqaqi S, Sbiti A (1996). Cytogenetic and epidemiological profiles of Down syndrome in a Moroccan population: a report of 852 cases. Singapore Med J.

[5] Morris JK, Alberman E, Mutton D, Jacobs P (2012). Cytogenetic and epidemiological findings in Down syndrome: England and Wales 1989-2009. Am J Med Genet A.

